# American Dental Society of Europe

**Published:** 1888-02

**Authors:** E. A. Galbreath

**Affiliations:** Hannover


### AMERICAN DENTAL SOCIETY OF EUROPE.

### FIFTEENTH ANNUAL MEETING AT COBLENTZ, GERMANY, SEPTEMBER, 1887.

Reported for the Independent Practitioner by Dr. E. A. Galbreath, Hannover.

Continued From Page 38.

### THURSDAY AFTERNOON SESSION CONTINUED.

*Dr. Elliot*—I desire to mention one valuable medicine which is not generally known, ethylate of sodium, a thick, syrupy liquid, which destroys hypertrophied gum without pain. It seems impossible to use it in excess, and it has never done any harm by going between the teeth. It was introduced by a Dr. Richardson, who is not a dentist.

*President George*—Will Dr. Förberg kindly explain how he grinds porcelain cavity blocks?

*Dr. Förberg*—I first shape the cavity, press a piece of paper capable of taking a sharp impression upon it, cut to the form indicated, stick it upon the selected porcelain tooth, and grind away all but the part covered by the paper. In this way the grinding can be done in a little time.

*President George*—Can some one report whether or not the Herbst process has been gaining ground?

*Dr. Miller*—It has not made much progress in Germany lately. At the last meeting of the German dentists it was not even mentioned. A year ago I sent letters of inquiry to a large number of German dentists, and they all answered that they had been obliged to make the fillings over. I have also heard from three or four parties who were enthusiastic over it, but who have now dropped it. I am certain that not one-tenth use it now of those who did two years ago.

*Dr. Rosenthal*—Had made extensive experiments, and had found that it was agreeable to the patients, but gave in general bad results.

*Dr. Patton*—Two years ago, at the meeting of the German Dentists of the Rhine and Westphalia, Dr. Herbst filled two proximal cavities, between two bicuspids, using a separating file as a matrix. He used the rotary method some, but hand pressure more. It took as long to make the fillings as it would have done by the hand alone. I examined the fillings with an excavator, and found the edges soft at several points, particularly at the neck. Dr. Herbst said that doing the work at a clinic, in a hurry and with strange surroundings, it was not so well done as at home.

*Dr. Förberg*—I had no intention to take the floor, yet I feel compelled to do so, in consequence of the severe criticism that Dr. Herbst, his method and appliances have been subjected to. I always thought it a fallacy to consider the rotary method wonderfully easy. Every one of you gentlemen had been working hard for years before he attained his present skill and perfectness of result. But now, any one who has seen some fillings made by rotation, or at the most made some few trials therewith, believes himself to be a master of the method. If he does not succeed, it is never he, but the method, that bears the blame. I believe it wants exactly the same accuracy and just as much skill as does any other method, if you wish to produce perfect results. I first use the gold unannealed (introducing and condensing it nearly as you used to do with soft gold), and finish with annealed foil. Dr. Herbst often uses heavy rolled gold, up to No. 200 or 300, for the surface. You can build up the contour only by the use of rotatory stone instruments, without matrix or hand pressure. So far as I can judge, the Herbst method unites the advantages of the methods for soft and for cohesive gold filling; it gives a most perfect adaptation, and the stone bur does not annoy the patient as much as the mallet. Yet it would be unwise to expect dentists who, for perhaps half their lifetime, had been using instruments and methods by the means of which they were able to perform the most excellent operations, now to throw overboard all that, in order to try something new instead. The objections urged against Herbst's ring-matrix are just as valid against all kinds of matrices. I find this matrix easy to adapt, and more practical in every respect than any other. For tin and gold fillings it can be so arranged as to leave just sufficient surplus material to condense afterwards, saving a good deal of unnecessary filing. The wedge-matrix was intended only for amalgam. The specimens on the table are, so to say, developments of the ring-matrix. Some of them were not new to American operators. The great thing, however, in Herbst's inventions (because with him they *were* original inventions), was the simple way in which he gained his results. Thus he made it possible for the great public to have the benefit of remedies and operations which before were obtainable only by the rich people.

*Dr. Miller*—I often find the teeth too close to enable me to use a matrix. I saw Dr. Herbst put one on in a clinic, and the patient wept. After seeing that, I have never had the courage to use one myself.

*Dr. Förberg*—Dr. Herbst has made a number of fillings for me. They are good, and I am satisfied. I have seen many of his patients, and they have been well treated.

*Dr. Jenkins*—A few years ago, Manfred was put on the stage of the Hoftheater in Dresden, as magnificently as it was possible to do it; and when it was all over the conclusion was that a country which had Faust had no use for Manfred. And so I would say that the man who knows how to use soft gold has no use for the Herbst method.

*Dr. Field*—That is quite true.

Dr. Tierney read a paper before the society, giving the history of an interesting case. A young lady of about eighteen presented herself with the ordinary symptom of alveolar abscess in the left superior first molar region, which appeared finally to involve the antrum. He extracted the molar and second bicuspid. The usual treatment and frequent cleansing seemed at first to cause an improvement, but after a week's time there appeared to have been no great advancement. He again examined the antrum with a probe, and found in it a hard substance which he removed, and now presented to the society for inspection. It was a temporary molar tooth, much absorbed and somewhat decayed. The question was, how did it get there, and what brought it into that condition ?

*Dr. Miller*—This tooth evidently was not in process of development, but fully grown. It has the appearance of a tooth in process of absorption, and therefore at these points it must have been in connection with living tissue. It also shows evidence of caries, which could not take place unless it were at least partially erupted.

*Dr. Tierney*—Could not the sharp edges have been produced by the dissolving of the tooth in pus ?

*Dr. Miller*—No.

*Dr. Kingsley*—I have seen a case somewhat like this. Perhaps the sixth and twelfth year molars, under abnormal conditions, might push a retarded temporary molar up into the jaw, and if so, why not into the antrum ?

*Dr. Miller*—I should hardly think it possible.

*President George*—We should like to hear something of Dr. Elliot's hand-piece for the engine.

*Dr. Elliot*—This hand-piece differs from all others in many particulars. First, you will see that the cable is enclosed in the French sheath, made of fine woven wire. The bur is held by a lock, and centered by a chuck. It is taken apart, as you see, in almost no time at all. By a spring movement it is released from the cable in a twinkling, thus allowing, as I am showing you, the right angle to be attached directly to the cable. The advantages of this will be apparent to all. I have also two pluggers which I attach directly to the cable. The blow is given by a rotating cam. Such an instrument must be run more rapidly than the usual machine admits of. One must have a power which will give three thousand revolutions per minute. I have here, also, some raw-hide polishing points, which I have found better than leather, some gutta percha points for filling roots, and some odorless oil.

Two years ago I put in a small gas engine for running the machines in my office and laboratory. Its maximum is about one horse power. Its minimum, about one cat power. It runs in the laboratory three lathes and a machine for drawing wire, and in the operating room, the usual machines. For the boring machine I have it arranged to give me three speeds; about seven hundred, fifteen hundred and three thousand revolutions per minute. The high speed is better for the dentist, but more disagreeable to the patient, so that I generally use a speed of eight hundred. It has cost me in consumption of gas two and one-half cents a day, and runs from eight to ten hours per day.

Dr. Abbott translated a letter from Dr. Hesse of the University of Leipzig.

Dr. Hesse held that it was not necessary for a German to go to America to acquire a dental education now. Formerly the professor made operations before the students, they looking on. That comprised their education. But three years ago Dr. Hesse himself took the first step in advance, and introduced the American system of clinical work. A year later it was introduced in the University of Berlin. He believes the attendance upon American colleges by German students is unwise, first, on account of the barrier of a foreign tongue preventing their proper understanding of the lecture, and second, because they lose valuable time. The only advantage that Dr. Hesse can see in a trip to America by the young German dentist, is that of seeing the many fine operators who are to be found in every American city.

(TO BE CONTINUED.)

